# BrainStereo: clinical application and efficiency evaluation of an open-source stereotactic planning tool

**DOI:** 10.1007/s00701-025-06564-x

**Published:** 2025-05-28

**Authors:** Zhongjie Shi, Dongfeng Li, Sifang Chen, Xin Gao, Hongjia Li, Xiyao Liu, Zhangyu Li, Zhanxiang Wang

**Affiliations:** 1 School of Medicine, Xiamen University, Xiamen, 361000 China; 2 The First Affiliated Hospital of Xiamen University, Xiamen, 361000 China; 3 Sanya Hospital of Traditional Chinese Medicine, Sanya, 572000 China

**Keywords:** Stereotactic, Leksell frame, Neurosurgery, Open-source toolkit

## Abstract

**Objective:**

To develop a flexible and open-source stereotactic surgical planning toolkit, validated through clinical data to assess its performance in frame registration and stereotactic neurosurgical planning.

**Methods:**

BrainStereo was developed based on the Leksell stereotactic frame principles and the 3D Slicer platform. It features an interactive interface for frame registration based on the custom-designed Layerwise Max Intensity Tracking (LMIT) algorithm, automated target/entry point calculation, and real-time 3D visualization. A retrospective analysis of stereotactic CT data from two hospitals was conducted, comparing BrainStereo with standard planning software to evaluate accuracy and efficiency.

**Results:**

BrainStereo was developed as a comprehensive toolkit integrating frame registration, target and entry point computation, and dynamic 3D visualization. A total of 86 CT datasets from two hospitals were included. The root mean square error (RMSE) for frame registration was 0.56 ± 0.23 mm. Computation time for BrainStereo was 5.54 ± 1.16 min, significantly longer than the standard toolkit (4.75 ± 0.83 min, 95% CI: 4.57–4.92 min, p = 0.001), but showed a steeper learning curve. The mean Euclidean distance between target points from both toolkits was 0.82 ± 0.21 mm (95% CI: 0.74–0.90 mm), with no significant differences along the X, Y, and Z axes. Entry point deviations were 0.47° ± 0.37° (p = 0.07 for arc and p = 0.06 for ring). Bland–Altman analysis confirmed strong agreement, supporting BrainStereo’s reliability for stereotactic neurosurgical planning.

**Conclusions:**

BrainStereo is an open-source stereotactic planning tool that provides neurosurgeons and researchers with a flexible alternative to proprietary software. Integrated within 3D Slicer, it allows for adjustable parameters and modular functionality, addressing some of the limitations commonly associated with commercial solutions, such as hardware restrictions and limited adaptability. By offering open-source access, BrainStereo fosters transparency, collaboration, and broader accessibility, potentially advancing the field of stereotactic neurosurgery.

**Supplementary Information:**

The online version contains supplementary material available at 10.1007/s00701-025-06564-x.

## Introduction

Stereotactic methods play a crucial role in neurosurgery, enabling precise targeting for the treatment of brain lesions, pathological biopsies involving intracranial structures, functional procedures such as deep brain stimulation (DBS) and stereoelectroencephalography (SEEG), as well as other high-precision interventions [18, 8, 23]. Among the available stereotactic systems, the Leksell stereotactic frame stands out as the most commonly utilized globally, praised for its dependability and versatility. [26, 10, 7, 24] Initially developed by Professor Lars Leksell in 1949, this system has set the benchmark for stereotactic surgery and continues to lead the field. [4, 5]

Stereotactic technology enables highly targeted and precise interventions in neurosurgery [25, 16, 12, 9]. However, most currently available stereotactic planning toolkit is commercially developed and tightly integrated with proprietary hardware, presenting several limitations [13, 20]. First, these commercial platforms are closed-source, restricting their use to specific operating environments while preventing users from accessing the source code or modifying functionalities to meet their specific needs. Second, different manufacturers’ planning toolkit often lacks interoperability, making it difficult to process data generated by other stereotactic systems and leading to poor cross-platform compatibility. Third, the slow iteration cycle of commercial toolkit can result in stagnation, limiting its ability to adapt to rapid advancements in medical technology and clinical practice. Additionally, the high cost of these commercial platforms poses a significant barrier to widespread adoption, hindering their accessibility, particularly in resource-limited settings. Consequently, existing solutions may fail to meet modern neurosurgical requirements.

To the best of our knowledge, there appears to be a lack of openly available or open-source stereotactic planning toolkits in the existing literature and major software repositories. The development of a flexible, open-source stereotactic toolkit could help offer valuable benefits not only for clinical surgical planning but also for education and training in neurosurgery.

In this work, we introduce BrainStereo, a versatile stereotactic planning toolkit designed to enable real-time tracking and visualization of both target and entry points. Unlike proprietary commercial systems, BrainStereo operates independently of specific platforms, offering customizable parameters for compatibility with stereotactic frames from various manufacturers. Full source code is publicly accessible, allowing users to freely download, modify, and tailor the toolkit for their clinical or research purposes.

## Methods

### Toolkit design and implementation

#### Design principles

**Fig. 1:**
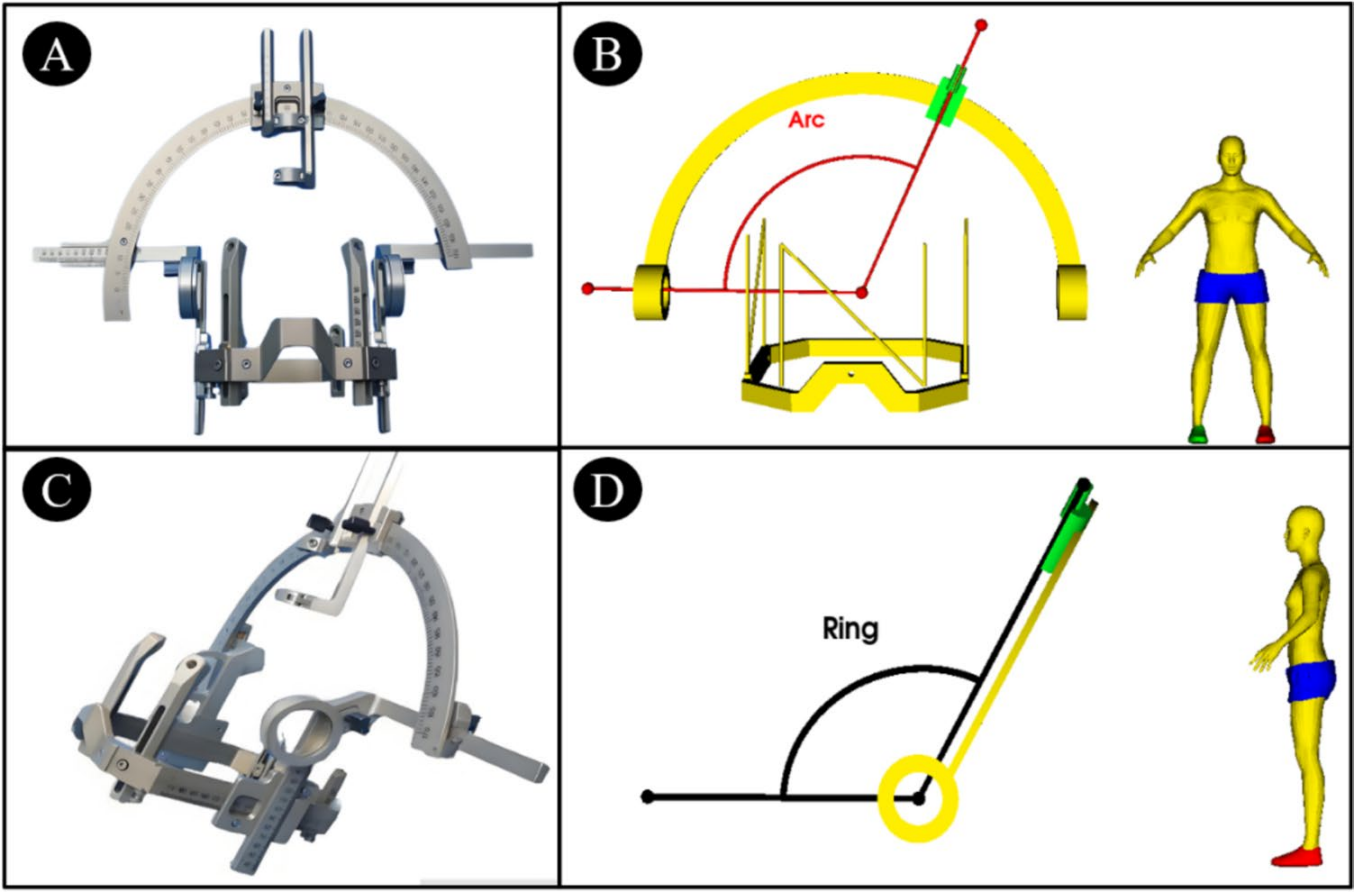
Structure of the Leksell stereotactic frame and illustration of arc and ring angles. (**A**) Anterior view of the Leksell stereotactic frame. (**B**) Demonstration of the arc angle on the Leksell frame model. (**C**) Lateral (left) view of the Leksell frame. (**D**) Demonstration of the ring angle on the Leksell frame model

The Leksell stereotactic system operates on the arc-center principle, incorporating a Cartesian coordinate system and a semi-circular arc as its core components. In the context of stereotactic surgery, the spatial coordinates within the Cartesian coordinate system are essential for accurately defining the location of the surgical target. Both arc and ring angles are crucial for describing the entry point’s position as well as the trajectory that connects the entry point to the target. The arc angle refers to the angle between the trajectory of the puncture and the left–right axis of the patient (Fig. 1A–B), while the ring angle refers to the angle between the puncture trajectory and the posterior-anterior axis of the patient (Fig. 1C–D).

**Fig. 2:**
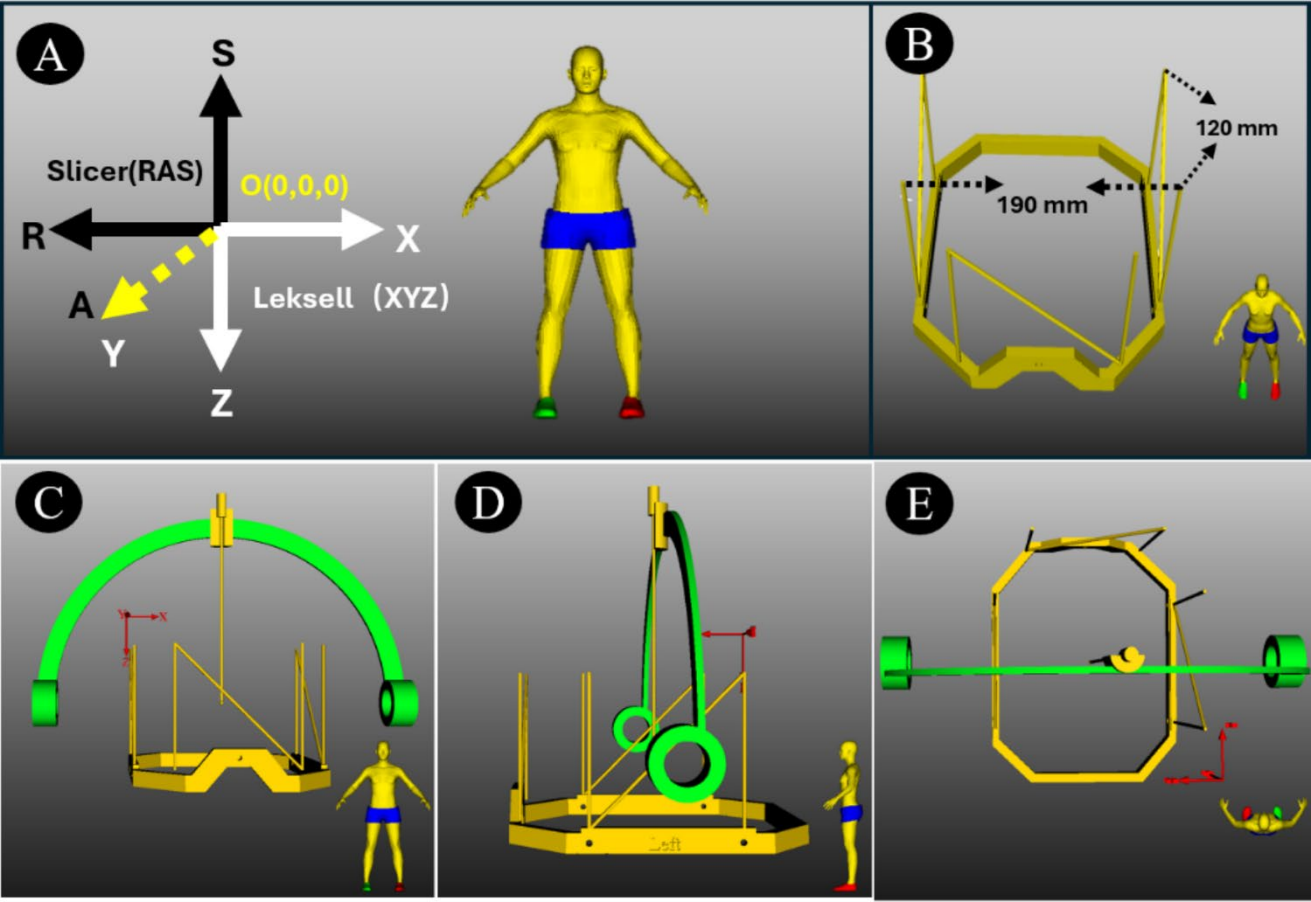
The RAS coordinate System and Leksell Frame model. (**A**) Comparison between the RAS and Leksell Cartesian coordinate systems, which share a common origin. The RAS system is shown in black, and the Leksell system in white. The yellow arrow indicates the axis oriented perpendicularly to the viewing plane, pointing from posterior to anterior. This axis corresponds to the A-axis in the RAS system and the Y-axis in the Leksell coordinate system. while the directions of the other two axes are reversed. (**B**) Dimensions of the Leksell stereotactic frame, including the overall width and the characteristic N-shaped fiducial board. (**C**-**E**) Orthogonal views of the Leksell frame: anterior (front), left lateral, and superior (top) perspectives

The RAS (Right, Anterior, Superior) coordinate system is a commonly used anatomical reference frame. It defines the spatial position of points relative to the patient’s body, with the origin typically located at the patient’s center, and the three axes pointing toward the right, anterior, and superior directions. Figure 2A illustrates the spatial relationship between two coordinate systems.

To make the Leksell frame coordinate system compatible with the RAS system in 3D Slicer, we performed a transformation on it [30, 6, 3]. Specifically, we calculated the coordinates of the target and entry points in the RAS coordinate system. Then, these coordinates are converted into values in the Leksell frame coordinate system. The core algorithm of the BrainStereo module is developed based on the geometric structure and mathematical principles inherent to the Leksell stereotactic frame.

#### Frame registration

Frame registration is a crucial step in stereotactic imaging, as it transforms imaging data into a standardized position necessary for accurate coordinate calculation. Only after completing frame registration can the mathematical model used in this study be applied to determine the precise coordinates of each point.

The Leksell stereotactic frame typically features two N-shaped fiducial boards on both lateral sides, each with dimensions of 120 mm in height and width. These fiducial boards form a rectangular structure with a distance of 190 mm between them, which is a commonly used parameter **(**Fig. 2B**)**. The frame’s center is defined as (100, 100, 100), with the origin (0, 0, 0) positioned at the superior posterior right corner. Consequently, the four vertices of the rectangular structure are: left anterior: (195, 160, 40); right anterior: (5, 160, 40); left posterior: (195, 40, 40); right posterior: (5, 40, 40).

To convert the Leksell image space coordinates to the 3D Slicer coordinate system, the frame’s center is translated to the RAS system origin (0,0,0). Additionally, the frame’s orientation is adjusted to align the Leksell coordinate axes (X, Y, Z) with the RAS coordinate axes (R, A, S) in the following manner:

- X-axis is aligned with the R-axis but in the opposite direction.
- Y-axis is aligned with the A-axis in the same direction.
- Z-axis is aligned with the S-axis but in the opposite direction.

The four vertices in the 3D Slicer coordinate system are computed as: left anterior: (−95, 60, 60); right anterior: (95, 60, 60); left posterior: (−95, −60, 60); right posterior: (95, −60, 60). Figure 2C-E show models of the Leksell head frame from different perspectives.

After registration, this standardized frame’s position is defined as the reference position. The process of transforming an arbitrary Leksell frame data into this reference position is termed frame registration.

**Fig. 3:**
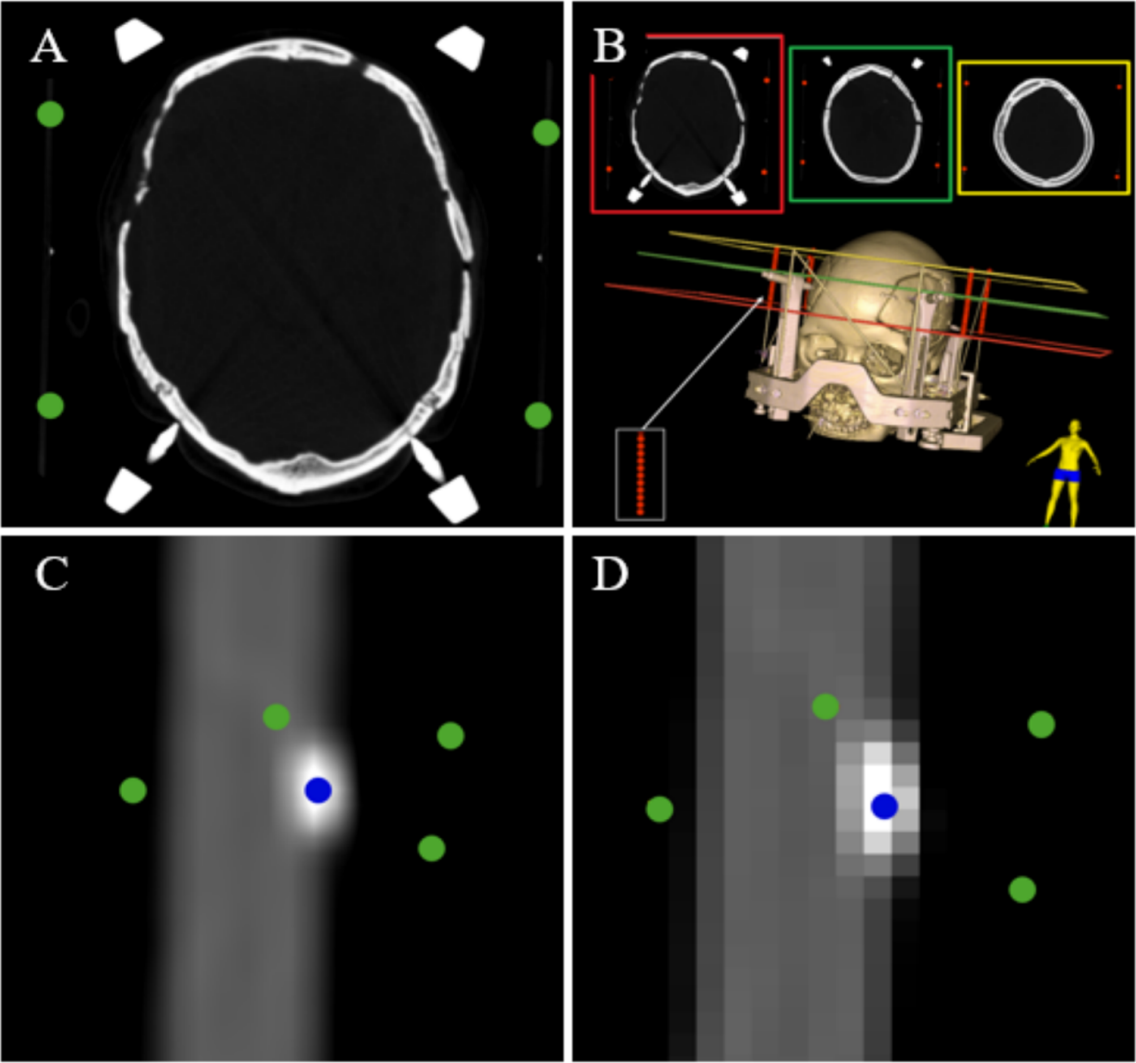
Vertex identification based on the LMIT algorithm. (**A**) Four roughly selected points (green) are placed on an arbitrary slice. (**B**) The algorithm then performs upward layer-by-layer tracking, automatically marking the path in red until the vertex is identified. (**C**) To simulate user variability, five sets of initial points (green) were assumed to reflect possible manual placement deviations. (**D**) The software expands a 10-pixel region around each point and automatically locates the voxel with the highest CT value, correcting each point to that location (blue)

To rapidly identify the four vertices in a new Leksell frame-based CT while maintaining user flexibility, we designed an algorithm called Layerwise Max Intensity Tracking (LMIT). The algorithm works as follows: the user manually selects four points on any axial slice, without considering the order. Around each selected point, a 10-pixel neighborhood is defined, and within this region, the voxel with the highest CT intensity is identified. The algorithm then scans sequentially through the slices starting from the layer where the points were placed, moving upward slice by slice, tracking the highest-intensity voxel at each step. The search continues until the maximum intensity drops below 500, at which point the tracking terminates. The final tracked position for each point is considered the target vertex, The entire process is highly efficient, completing within 0.5 s. Although the initial four points are manually selected, this approach significantly reduces subjective error by relying on intensity-based tracking rather than user judgment. This ensures consistency and accuracy in vertex identification. **(**Fig. 3**)**.

Once the four target vertices are determined, the Kabsch algorithm [14] is used to compute the optimal rigid transformation matrix that aligns these points with the predefined reference points in the 3D Slicer coordinate system, enabling rapid and precise frame registration.

#### Target calculation

Based on the above theory, once the frame registration is completed, the coordinate transformation between the 3D Slicer image space and the Leksell frame coordinate system is established using the following formula:

$$(X,Y,Z)=\left(100-R,100+A,100-S\right)$$

where (R,A,S) represents the target coordinates in 3D Slicer and (X,Y,Z) represents the target coordinates in Leksell frame system.

#### Entry calculation

In 3D Slicer, let the target coordinates be represented as Target (Tr, Ta, Ts) and the entry point coordinates as Entry (Er, Ea, Es). The vector from the target to the entry point, denoted as Ver_TE, is then defined as the line connecting these two points.

The arc angle is computed as the angle between Ver_TE and the R-axis (X-axis in the Leksell frame) using the inverse cosine function:

$$\text{arc}=\left|{\text{cos}}^{-1}\left(\frac{{E}_{r}-{T}_{r}}{\sqrt{({E}_{r}-{T}_{r}{)}^{2}+({E}_{a}-{T}_{a}{)}^{2}+({E}_{s}-{T}_{s}{)}^{2}}}\right)\right|\times \frac{180}{\pi }$$

The ring angle is determined by the projection of Ver_TE onto the A-S plane (Y–Z plane in Leksell coordinates) and its angle relative to the A-axis (Y-axis in the Leksell frame), computed using the inverse tangent function:

$$\text{ring}={90}^{\circ }-\left(\text{arctan}\left(\frac{{E}_{a}-{T}_{a}}{{E}_{s}-{T}_{s}}\right)\times \frac{180}{\pi }\right)$$

Using the above formula, the target and entry point coordinates based on the Leksell frame coordinate system can be calculated. However, to align with the markings on the frame, minor adjustments may be necessary in practical applications, though these adjustments are not mandatory.

#### Dynamic 3D visualization

To make the surgical planning more intuitive and not limited to abstract numerical data, we designed a comprehensive 3D model that includes the semi-circular arc, trajectory channel, and stereotactic frame. Throughout the surgical planning process, these models dynamically adjust their positions in real-time, simulating the actual frame orientation used in a live surgical procedure. All the models are integrated within BrainStereo.

### Clinical data validation

#### Data collection and processing

This study retrospectively collected CT imaging data from patients who underwent stereotactic frame-based localization at the First Affiliated Hospital of Xiamen University and Sanya Traditional Chinese Medicine Hospital between February 2023 and December 2024. All patients underwent helical CT scanning using a 64-slice Siemens CT scanner (SOMATOM, Forchheim, Germany) while wearing a Leksell-type stereotactic frame (model ASA-602S, manufactured by Anke, China). Scanning parameters were as follows: slice thickness of 1 mm, contiguous slices, matrix size of 512 × 512, field of view (FOV) approximately 240 mm, tube voltage 120 kVp, and tube current ranging from 200 to 300 mA. All images were exported in DICOM format for subsequent 3D reconstruction and image registration analysis.

**Inclusion Criteria:**

- High-quality imaging with minimal artifacts around the lesion.
- Use of the Leksell stereotactic frame, with clear visualization in the images.
- Submillimeter slice thickness (≤1 mm).

**Exclusion Criteria:**

- Poor image quality with excessive artifacts.
- Incomplete imaging data, such as missing slices.

All qualifying CT datasets underwent anonymization and were assigned random numbers prior to additional processing steps. These datasets were subsequently imported into the 3D Slicer software. A senior neurosurgeon with 25 years of clinical surgical experience collaborated with a neurosurgical resident who holds a PhD in a medical-engineering interdisciplinary program. His doctoral research focused on image-guided surgery. He is highly proficient in the use of 3D Slicer, capable of independently performing all essential operations and developing customized modules within the platform. Importantly, neither of these two physicians was involved in the original surgeries included in this study, and they had no prior exposure to the anonymized CT datasets. For each CT scan, a target point was determined at the center of the lesion. This was followed by the planning of the surgical trajectory and the selection of the entry point on the skin surface. To enhance the precision of target point localization during the testing phase and to minimize subjective bias in point selection, a voxel filling technique was employed within the CT images. Specifically, a spherical area with a diameter of 5 mm surrounding the target point was filled using a value of 1200 Hounsfield units (HU). Finally, the processed images were exported in DICOM format for further analysis.

#### Target and entry calculation

A young neurosurgeon with no prior experience using any stereotactic planning system, and who was not involved in the original surgeries of these patients and thus had no prior knowledge of the cases, performed target and entry calculations on the processed data using both BrainStereo and the standard, medically certified stereotactic toolkit for comparison. Prior to the task, the physician received a 20-min training session on each toolkit. For each dataset, the following parameters were recorded:

- Processing time: Measured from data import to completion of calculations.
- Target: Recorded as Leksell frame coordinates.
- Entry: Defined by arc and ring values.
- A different independent physician recorded all data to ensure consistency.

#### Ethics approval and consent to participate

This study was approved by the Institutional Review Board (IRB) of the First Affiliated Hospital of Xiamen University (Approval No. KYLS2021038), with additional approval obtained from the IRB of Sanya Hospital of Traditional Chinese Medicine (Approval No. KYLL2024-07).

### Statistical analysis

All statistical analyses were performed using SPSS 26.0. Paired t-tests were conducted to compare calculation time and accuracy between the two methods. Results were reported as mean ± standard deviation (mean ± SD), with statistical significance set at p < 0.05. Bland–Altman analysis was carried out to calculate the 95% limits of Entry’s agreement. `

## Results

### Toolkit development

The toolkit was successfully developed, integrating frame registration, target and entry point computation, and dynamic 3D visualization. The root mean square error (RMSE) for both frame registration and target/entry point localization is automatically displayed on the interface.

### Clinical results

#### CT image data

A total of 86 eligible patient datasets from two hospitals were included in the study. These comprised 33 cases of intracerebral hemorrhage, 24 cases of DBS, 21 cases of pathological biopsy, 5 cases of vascular malformation, 3 cases of external ventricular drainage (EVD). A pair of target and entry points was defined based on the characteristics of the lesion, resulting in a total of 86 target pairs.

#### Computation time

**Fig. 4:**
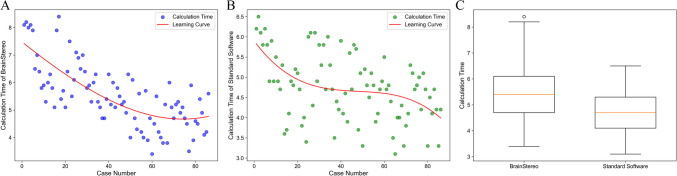
Comparison of calculation time between two methods. (**A**) Scatter plot of calculation time and quadratic regression analysis for BrainStereo. (**B**) Scatter plot of calculation time and quadratic regression analysis for the standard software. (**C**) Box plot comparing the calculation times of BrainStereo and the standard software, showing that BrainStereo takes longer on average

The computation time for BrainStereo was 5.54 ± 1.16 min (95% CI: 5.29–5.78 min), with a range of 3.40 to 8.40 min and a median of 5.40 min. In contrast, the computation time for the standard toolkit was 4.75 ± 0.83 min (95% CI: 4.57–4.92 min), with a range of 3.10 to 6.50 min and a median of 4.70 min. A paired t-test analysis showed that the difference in computation time between the two toolkits was statistically significant (95% CI: 0.57–1.01, t = 7.24, p = 0.001), indicating a substantial time difference between the two methods. Furthermore, quadratic regression analysis of both toolkits revealed a learning curve with a gradual reduction in computation time as the number of cases increased. However, the learning curve for the BrainStereo in this study was more pronounced, indicating a faster mastery compared to the standard software **(**Fig. 4**)**.

#### Frame registration accuracy

**Fig. 5:**
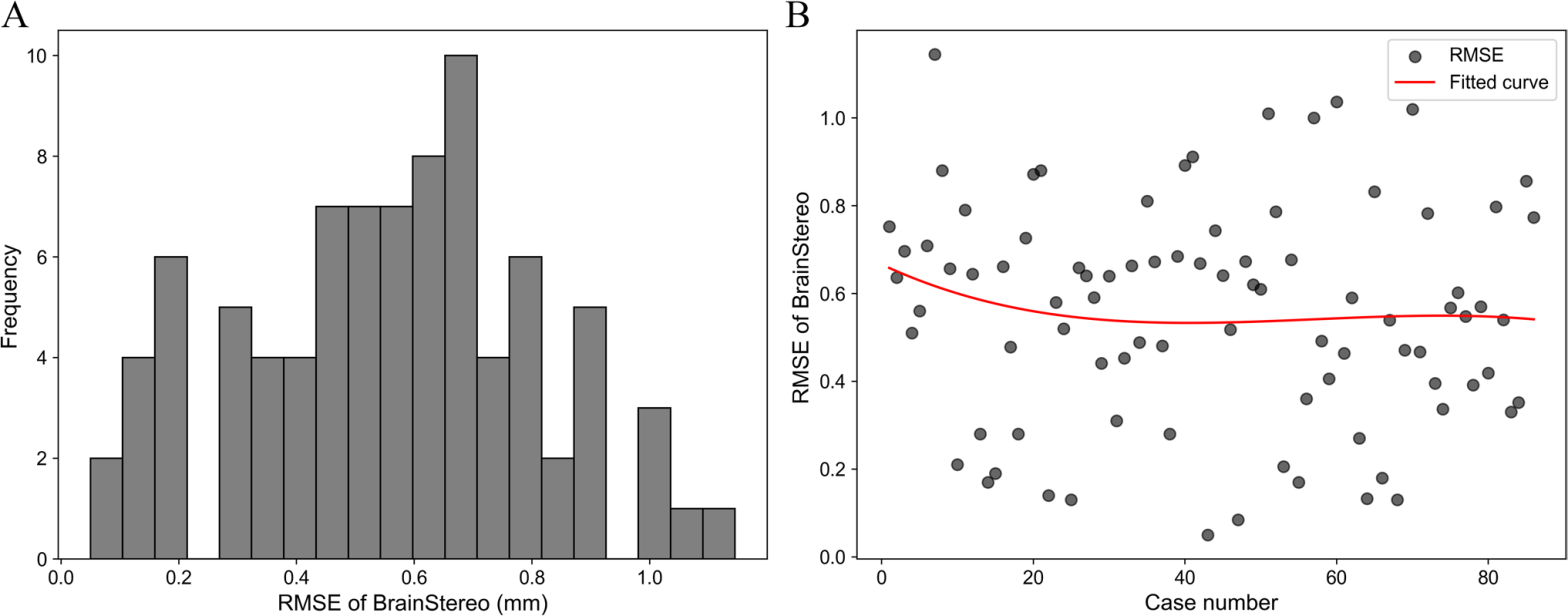
Frame registration accuracy in BrainStereo. (**A**) Frequency distribution of the root mean square error (RMSE) in frame registration using BrainStereo. (**B**) Scatter plot of RMSE across different cases, with a fitted trend line shows that the RMSE remains relatively stable across different cases

For the 86 datasets, the RMSE for frame registration ranged from 0.05 to 1.14 mm (95% CI: 0.50–0.61 mm), with a mean of 0.56 ± 0.23 mm and a median of 0.58 mm. As the histogram indicates a normal distribution, approximately 68% of the RMSE values for the 86 datasets fall within the range of the mean ± one standard deviation, specifically between 0.33 mm and 0.79 mm. This shows that the majority of the frame registration accuracies are concentrated around the mean value, with relatively fewer data points in the tails of the distribution**(**Fig. 5**)**.

#### Target point accuracy

**Fig. 6:**
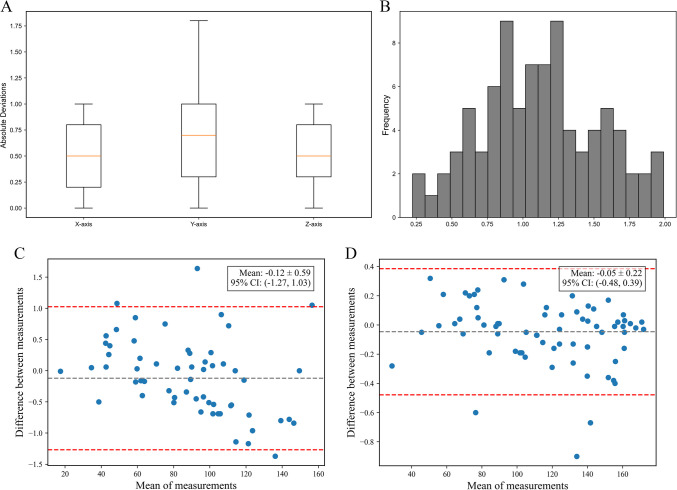
Target and entry accuracy between two ways. (**A**) Box plot comparing the absolute deviations of BrainStereo and standard software along the X,Y and Z axis. (**B**) Histogram of the Euclidean distances between the calculated target points of BrainStereo and those of the standard software. (**C**) Results of two software based on arc. (**D**) Results of two software based on ring

The average Euclidean distance between target points from the two toolkits was 0.82 ± 0.21 mm (95% CI: 0.74–0.90 mm), with a median of 0.74 mm **(**Fig. 6A–B). The absolute deviations along each axis were as follows:

- X-axis: 0.51 ± 0.33 mm (t = −0.71, p = 0.48), no significant difference
- Y-axis: 0.65 ± 0.27 mm (t = 0.73, p = 0.47), no significant difference
- Z-axis: 0.50 ± 0.29 mm (t = −0.55, p = 0.58), no significant difference

#### Entry point accuracy

The polar coordinates (arc and ring angles) of the entry point were compared between the two toolkits. The absolute deviations were 0.47° ± 0.37° for arc (95% CI: 0.39–0.55°, t = –1.91, p = 0.07; median = 0.43°) and 0.15° ± 0.17° for ring (95% CI: 0.12–0.19°, t = –1.93, p = 0.06; median = 0.12°), with no statistically significant differences. Bland–Altman analysis demonstrated high agreement between the toolkits: the arc angle difference was –0.12° ± 0.59° (95% limits of agreement: –1.27° to 1.03°), and the ring angle difference was –0.05° ± 0.22° (95% limits: –0.48° to 0.39°). These results indicate consistent and accurate entry point localization by both toolkits, with negligible bias (Fig. 6C–D).

## Discussion

### Stereotactic techniques

Stereotactic techniques are broadly divided into frame-based and frameless approaches. Research by Girgis et al. [10] has shown that in SEEG, frame-based methods outperform frameless navigation-guided systems in both accuracy and precision. This advantage likely stems from the rigid frame’s ability to constrain head movement, whereas frameless systems are more susceptible to intraoperative navigation errors. Given that SEEG electrode placement demands submillimeter precision (typically target errors < 0.8 mm) to accurately capture epileptogenic zone activity, frame-based techniques remain the gold standard [2, 31]. Their dominance persists in high-precision procedures, where submillimeter accuracy is essential due to the small size and critical proximity of targets such as the subthalamic nucleus or epileptic foci [19]. In addition, Wang et al. [27] introduced a method for treating intracerebral hemorrhage using a 3D-printed puncture guide. This approach does not rely on complex stereotactic frame, making it suitable for use in resource-limited settings. However, the dependence on 3D printing results in a relatively time-consuming preparation process, and once the surgical trajectory and target are determined, they cannot be adjusted intraoperatively. Yuan et al. [28] proposed a low-cost alternative to stereotactic techniques based on laser-guided positioning, in which preoperative imaging is combined with intraoperative laser alignment to guide the puncture trajectory. Nevertheless, this technique requires complete immobilization of the patient’s head during the procedure, and the laser path is susceptible to obstruction by surgical instruments or personnel, compromising intraoperative visibility and targeting accuracy.

### Frame registration accuracy

Leksell stereotactic frame registration differs fundamentally from complex multimodal image registration by relying on a rigid fiducial framework. In this study, alignment was performed by manually selecting four fiducial points, followed by automated registration, with root mean square error (RMSE) displayed in real time to allow adjustments when exceeding 1 mm. BrainStereo achieved a consistent RMSE of 0.56 ± 0.23 mm, outperforming the minimum automatic registration accuracies of 0.6–0.7 mm and 0.88–2.13 mm reported in prior studies [21, 22] Notably. RMSE showed no downward trend with increased usage, as the LMIT algorithm's automatic calibration of manually selected points significantly reduced subjective error, ensuring consistent accuracy.

### Target and entry localization

For target localization, the mean Euclidean distance between coordinates identified by two methods was 0.82 ± 0.21 mm, indicating a high degree of precision. This accuracy is sufficient to meet the precision requirements for DBS and other stereotactic applications.

For entry point localization, although the differences did not reach statistical significance (p = 0.07 for Arc and p = 0.06 for Ring), the observed values suggest a potential trend. Given the small absolute deviations, these differences are unlikely to have meaningful clinical implications. Future studies with larger datasets may help clarify whether these trends become statistically significant. In addition, The Bland–Altman analysis demonstrated strong agreement, reinforcing BrainStereo’s reliability and potential clinical applicability in stereotactic neurosurgical planning.

### Calculation time

Significant differences in computation time were observed between the two methods. The computation time for BrainStereo was 5.54 ± 1.16 min, compared to 4.75 ± 0.83 min for the standard software (t = 7.24, p = 0.001). BrainStereo required more time, and this difference can be attributed to two factors. First, BrainStereo utilizes a semi-automated registration process based on manual fiducial point selection, which inherently takes longer than the fully automated registration process used by the standard toolkit. Second, the experimental design played a role: after initially using BrainStereo to process the data, the neurosurgeon became familiar with the target and entry point positions. As a result, he was able to quickly locate these points when using the standard toolkit, thus reducing the computation time. This time difference suggests that future work should focus on optimizing the manual point selection process. Enhancements to maintain robustness while improving computational speed could lead to more efficient usage, particularly in clinical settings [11]. Although BrainStereo has a relatively longer average computation time, the observed steeper learning curve suggests that users can significantly improve their efficiency with increased experience. This may be attributed to its structured workflow and focused functionalities, which help reduce unnecessary complexity during the planning process. From a clinical perspective, this indicates that even users without prior experience can become proficient after a limited number of uses. These features collectively reflect a user-friendly design that facilitates rapid learning and application in clinical settings. Future studies incorporating objective performance metrics and user feedback—such as usability questionnaires and workflow assessments—will be valuable in clarifying training requirements and further optimizing clinical integration.

### Rationale for choosing 3D slicer

Popular open-source medical imaging platforms include 3D Slicer, MITK, ITK-Snap, and CustusX [29, 1, 15]. 3D Slicer was chosen as the foundation for BrainStereo due to its active community, extensive modular ecosystem, and broad applicability in medical imaging processing. Compared to MITK, which is C + + -based and requires significant development expertise for customization, 3D Slicer offers a more flexible Python-based scripting environment, making it easier for researchers and clinicians to extend its functionality. Additionally, ITK-Snap and CustusX, while useful for specific applications, have relatively limited functionality—ITK-Snap is primarily designed for image segmentation, whereas CustusX focuses on image-guided interventions but lacks comprehensive stereotactic planning tools.

BrainStereo, a Leksell frame-based planning tool integrated into the 3D Slicer platform, builds on these strengths. By refining frame registration and target localization, BrainStereo streamlines workflow efficiency while preserving the precision inherent to frame-based stereotactic surgery. Analysis of 86 stereotactic CT datasets underscores its clinical feasibility, demonstrating its potential to enhance stereotactic neurosurgical planning.

Additionally, BrainStereo inherits 3D Slicer’s open-source flexibility, allowing users to access and modify the source code, integrate custom algorithms, and seek community-driven solutions for specific clinical and research challenges. This feature overcomes the proprietary restrictions of commercial stereotactic planning toolkit, enhancing cross-institutional compatibility and fostering collaboration within the neurosurgical community. Additionally, the open-source nature of BrainStereo improves technical transparency and reproducibility, promoting interoperability across different software toolkits.

### Compatibility and cost-effectiveness

Unlike proprietary stereotactic planning systems, which are often expensive and require complex licensing, BrainStereo is free and compatible across multiple operating systems (Windows, macOS, and Linux), making it accessible to resource-limited healthcare institutions. Furthermore, BrainStereo supports CT and MRI datasets from different manufacturers, addressing the interoperability limitations of commercial toolkit.

The current version of BrainStereo introduces two adjustable parameters—frame fiducial width and height (default: 190 mm × 120 mm)—allowing compatibility with different Leksell frame models. This cross-manufacturer compatibility makes BrainStereo a cost-effective alternative to commercial systems, particularly for clinics and research facilities with limited budgets or those requiring greater flexibility.

### Efficient workflow

Leveraging the advanced 3D visualization capabilities of 3D Slicer, BrainStereo provides real-time and intuitive representations of the surgical trajectory, anatomical structures, and target/entry point positions. This feature facilitates preoperative simulation and optimization, which is particularly valuable for deep-seated brain lesions, such as those in the basal ganglia or thalamus. By offering an enhanced spatial understanding of complex anatomical relationships, BrainStereo helps neurosurgeons reduce intraoperative risk. With an intuitive step-by-step process-including target and entry point selection, automatic angle and coordinate calculation—users can rapidly complete surgical planning. This efficiency helps reduce preoperative preparation time and enhance clinical productivity, making BrainStereo particularly valuable in time-sensitive procedures, such as acute intracerebral hemorrhage or emergency stereotactic interventions. Although BrainStereo did not outperform commercial toolkit in terms of processing time in this study, its open-source and expandable nature allows for continuous workflow optimization and increased automation, making it progressively more efficient and intelligent over time.

### Limitations and future directions

Although BrainStereo demonstrates certain practical potential, this study has several key limitations. First, the study is based on a limited sample, which lacks statistical representativeness and persuasive power. Second, BrainStereo's performance was compared only with one commercial software, and this single dimensional evaluation cannot fully reflect its relative advantages. In the context of current commercial stereotactic systems, which are highly integrated and have mature workflows, it is still unclear whether BrainStereo can be considered comparable, and further rigorous validation is needed. Third, the evaluation of usability and learning curve was conducted by a single junior neurosurgeon. While this setup allowed for a preliminary assessment under standardized conditions, it introduces potential user bias and limits the generalizability of the findings. Differences in experience, interpretation, and operational habits among users may significantly influence performance. Future studies should involve multiple evaluators with varying levels of expertise to better assess inter- and intra-rater variability.

Therefore, based on the current evidence, BrainStereo cannot yet be considered an equivalent alternative to commercial systems, nor should its technical performance be regarded as superior. Its potential value is more likely to lie in providing a cost-effective, basic solution for specific contexts, such as resource-limited environments, and for clinical teaching practices [17]^.^ Future studies should adopt more rigorous designs, include a wider variety of surgical types, more extensive comparator systems, multiple readers, and clearly defined performance metrics to systematically validate its clinical practicality and limitations.

## Conclusions

In this study, we developed and introduced BrainStereo, an open-source stereotactic planning toolkit designed specifically for Leksell frame-based procedures. Based on the current limited sample, preliminary analysis suggests that BrainStereo achieves localization accuracy comparable to commercial tools, while offering real-time 3D visualization and an intuitive, expandable, and user-friendly interface.

### Software disclaimer

BrainStereo is an open-source software tool intended solely for research and educational purposes. It has not obtained any medical certification and is not approved for clinical use in surgical decision-making. The software is released under the MIT License, which permits free use, modification, and distribution, provided that the original copyright notice is retained. It is not a substitute for professional medical advice or clinical judgment. The responsibility for all surgical decisions rests entirely with the operating neurosurgeons. Prior to any clinical deployment, BrainStereo must undergo comprehensive regulatory evaluation and comply with relevant medical device and software standards.

## Supplementary Information

Below is the link to the electronic supplementary material.

Supplementary file1 (PDF 1069 KB)

## Data Availability

Data Availability: The complete code and all frame models for BrainStereo toolkit is publicly available at Github(https://github.com/xmszj/BrainStereo). Users can access, modify, and utilize the software for research.
