# Supplementary material for: BrainStereo: clinical application and efficiency evaluation of an open-source stereotactic planning tool
Source: Acta Neurochir (Wien). 2025 May 28;167(1):156. doi: 10.1007/s00701-025-06564-x (PMC12119382; doi:10.1007/s00701-025-06564-x)

Supplementary Manual

Basic Operation Guide for BrainStereo Software

1. Program Download and Installation

1.1 Download and Install the Latest Version of 3D Slicer（https://download.slicer.org）. If you are new to 3D Slicer, we recommend taking some time to explore its basic features. The official website offers comprehensive resources, and there is an active community available to assist with any questions or challenges .

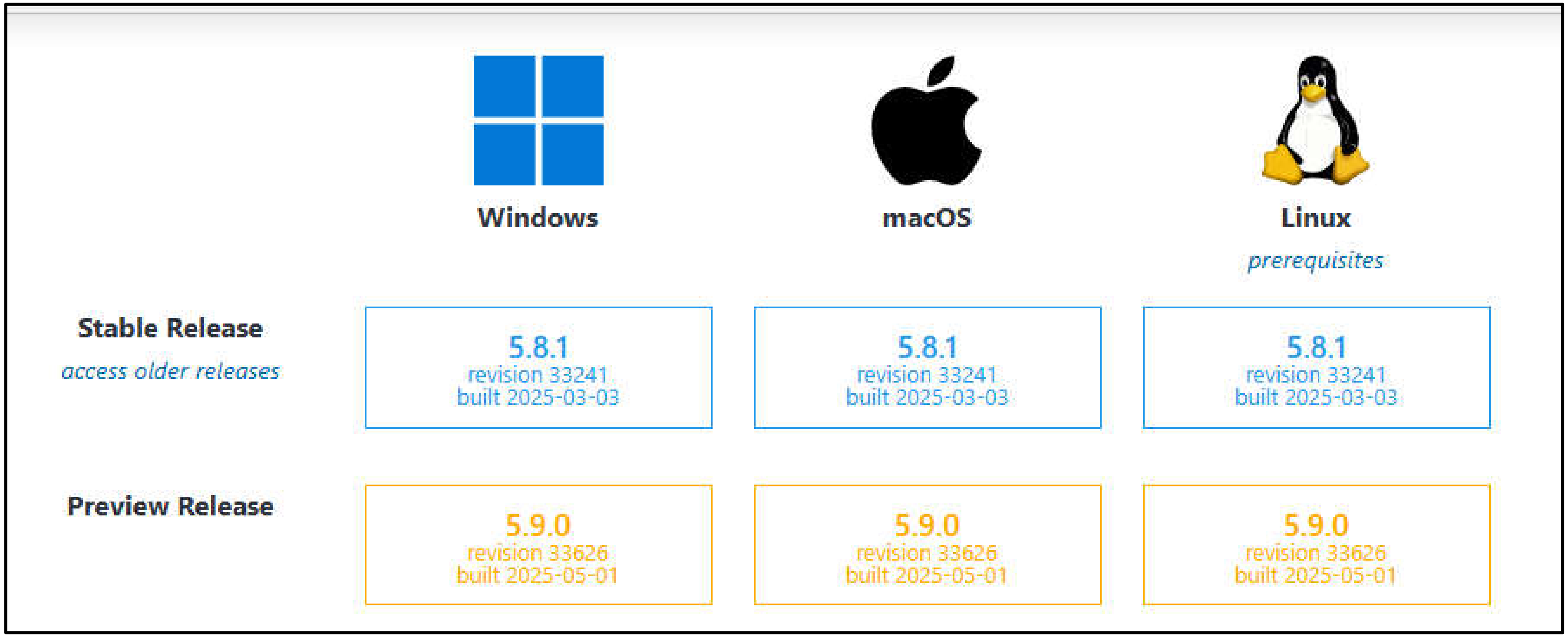

1. Program Download and Installation

1.2 Download the BrainStereo module from the repository: https://github.com/xmszj/BrainStereo . After extraction, place the entire module folder into a fixed directory—preferably within the installation path of 3D Slicer for easy access.The module includes the main algorithm script “BrainStereo.py ” and a “Resources” folder containing essential files such as the frame model and sample CT datasets.Please ensure the folder structure is preserved as shown below:

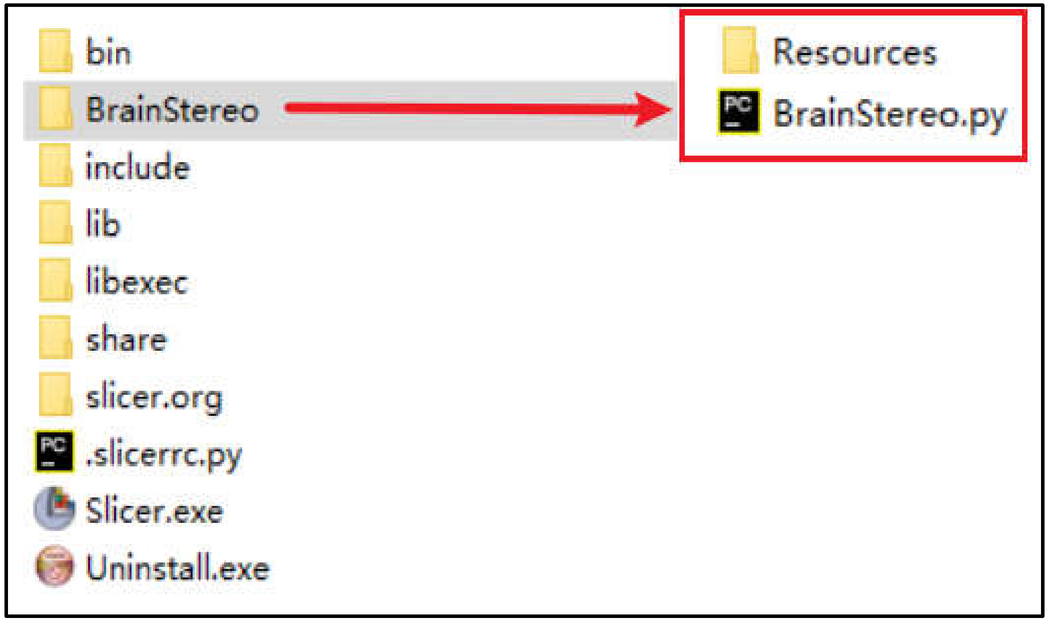

1. Program Download and Installation

1.3 Double-click ***Slicer.exe*** to launch the 3D Slicer application. Navigate to ***Edit*** → ***Application Settings*** → ***Modules***, then locate the ***Additional module paths*** section. Click the ***Add*** button and add the path of BrainStereo module directory. Restart 3D Slicer to complete the module registration. An illustration of this process is shown below:

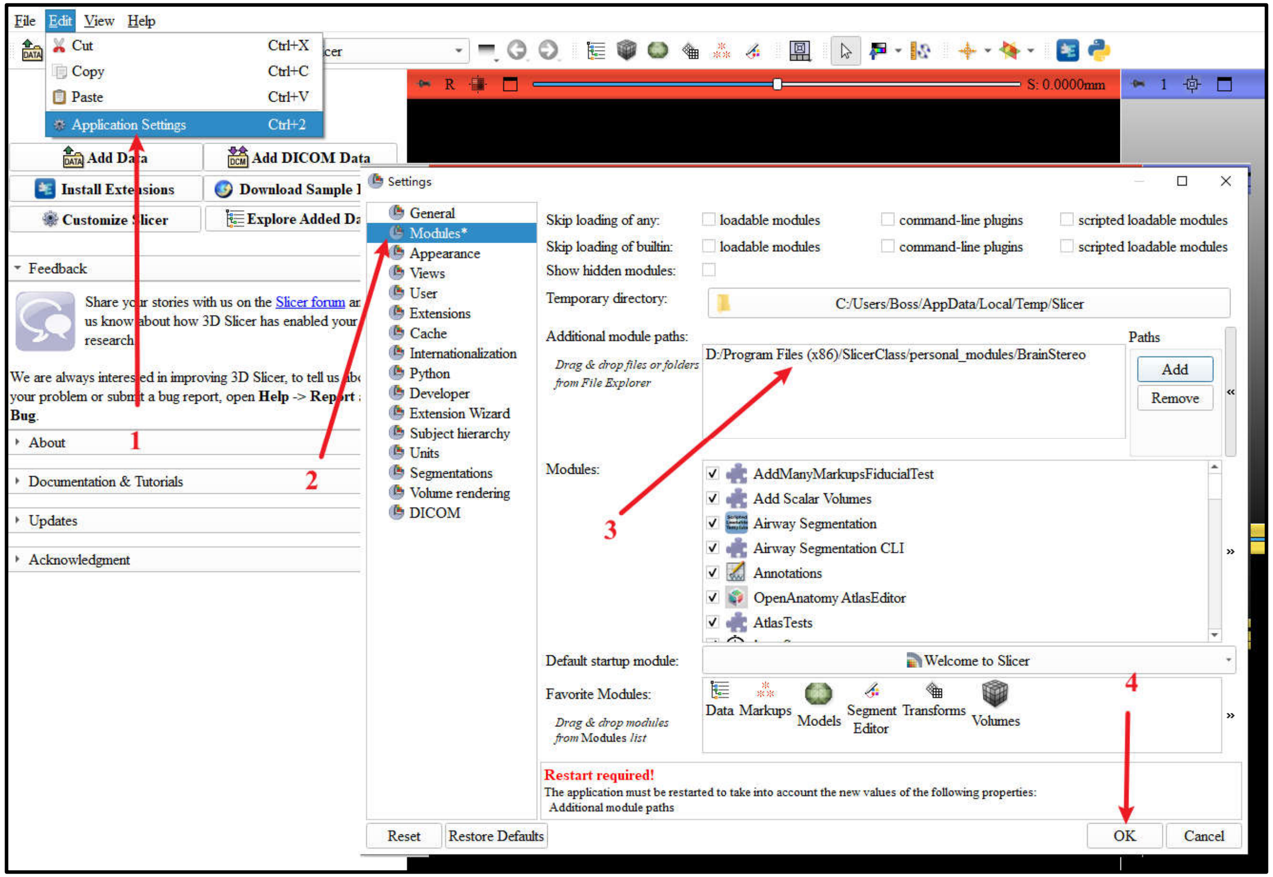

2、Module Usage

2.1 In the module ***search*** bar, type “brain” and double-click ***BrainStereo*** to launch the main interface of the program, as shown below:

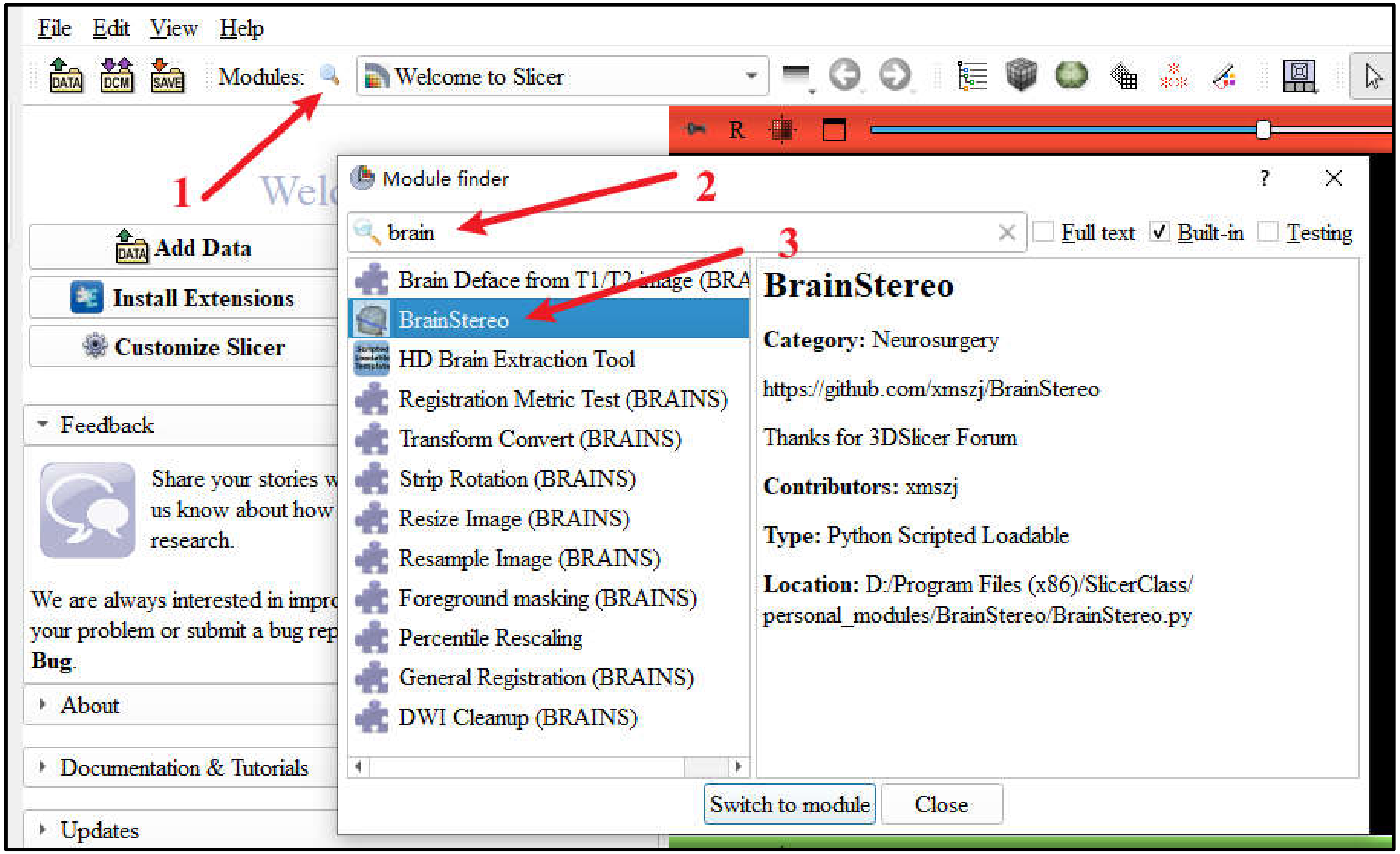

2、Module Usage

2.2 In the main interface of the program, click the ***Test*** button to load the pre-stored CT test dataset. Once the data is loaded, click the ***Show 3D*** button to automatically render and display the current CT in the 3D view. The green area (***Workflow***) corresponds to the head frame registration functionality, the purple area (***Result***) is for displaying the computational results, and the yellow area (***Visualization***) is dedicated to 3D visualization. Refer to the image below:

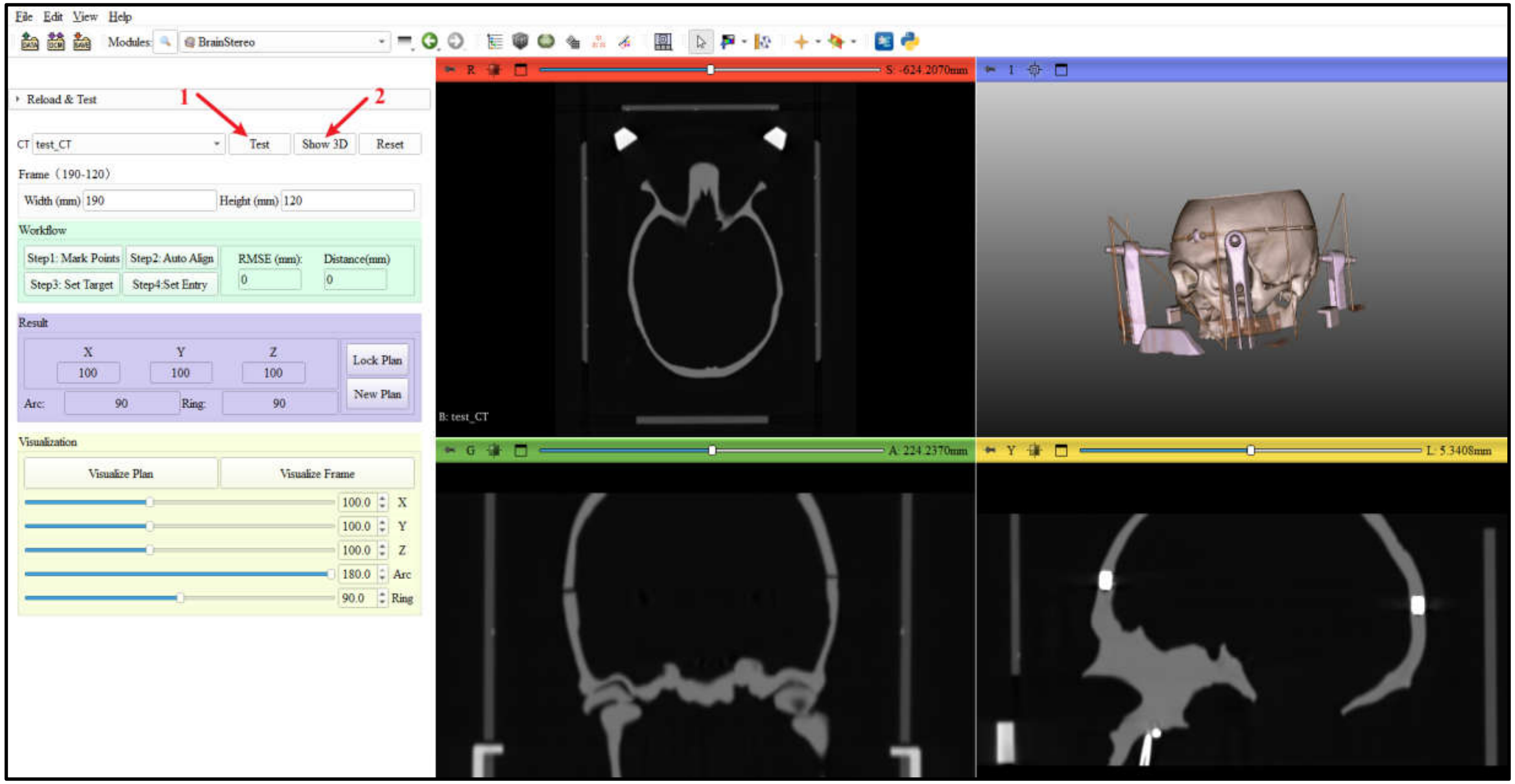

2、Module Usage

2.3 In the Workflow section, first click on ***Step 1: Mark Points***. Then, in the current axial view, mark the four points of the N-shaped reference board. The order of marking does not matter. Once all four points are marked, the software will automatically recognize them as points a, b, c, and d, and will correct the positions of the points based on the user's placement.

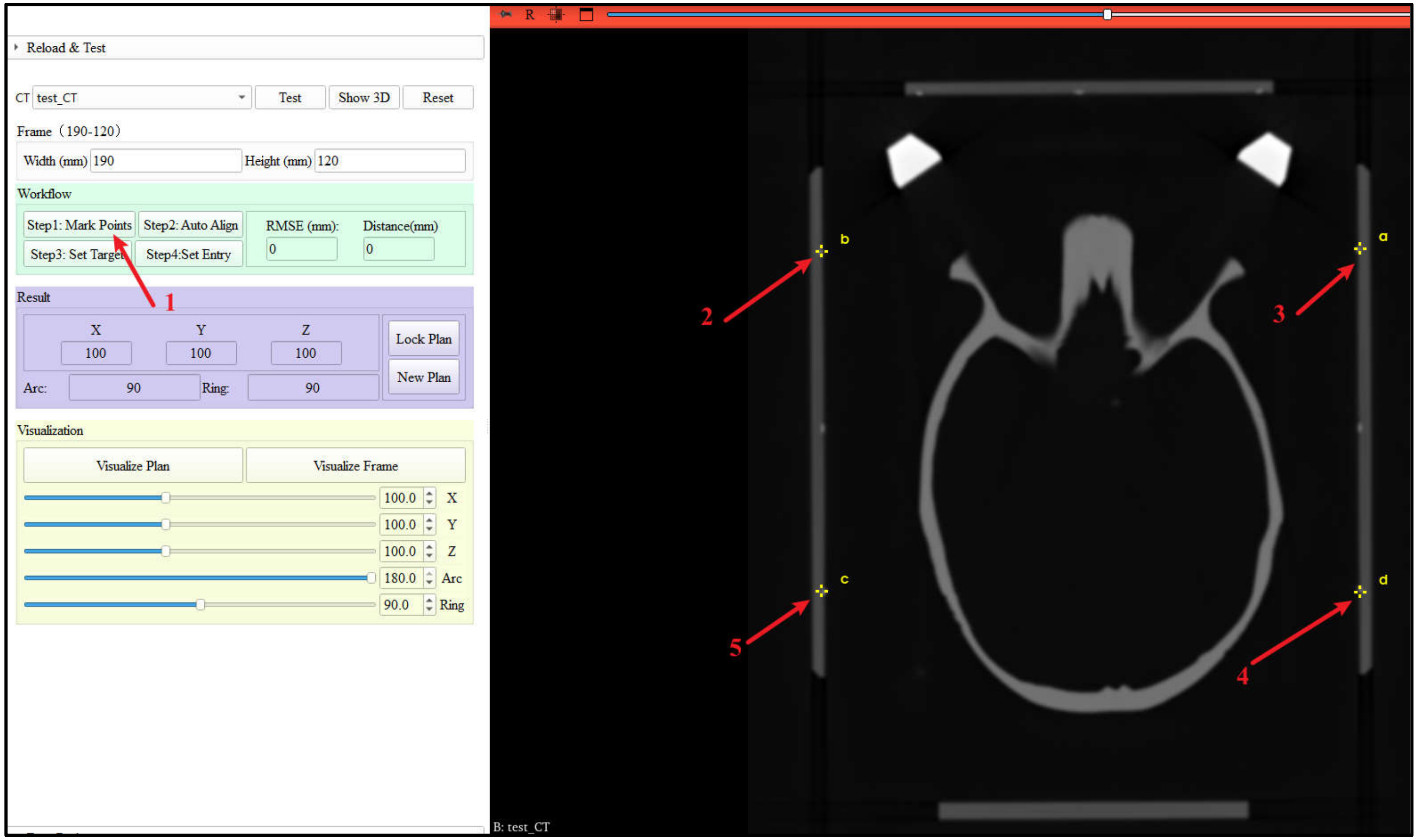

2、Module Usage

2.3 After completing Step 1, click on ***Step 2: Auto Align*** to perform the head frame registration calculation. The ***RMSE*** (Root Mean Square Error) displayed represents the registration accuracy, with units in millimeters. At this point, clicking on ***Visualize Plan*** allows for visual inspection of the registration accuracy between the current CT data and the reference head frame.

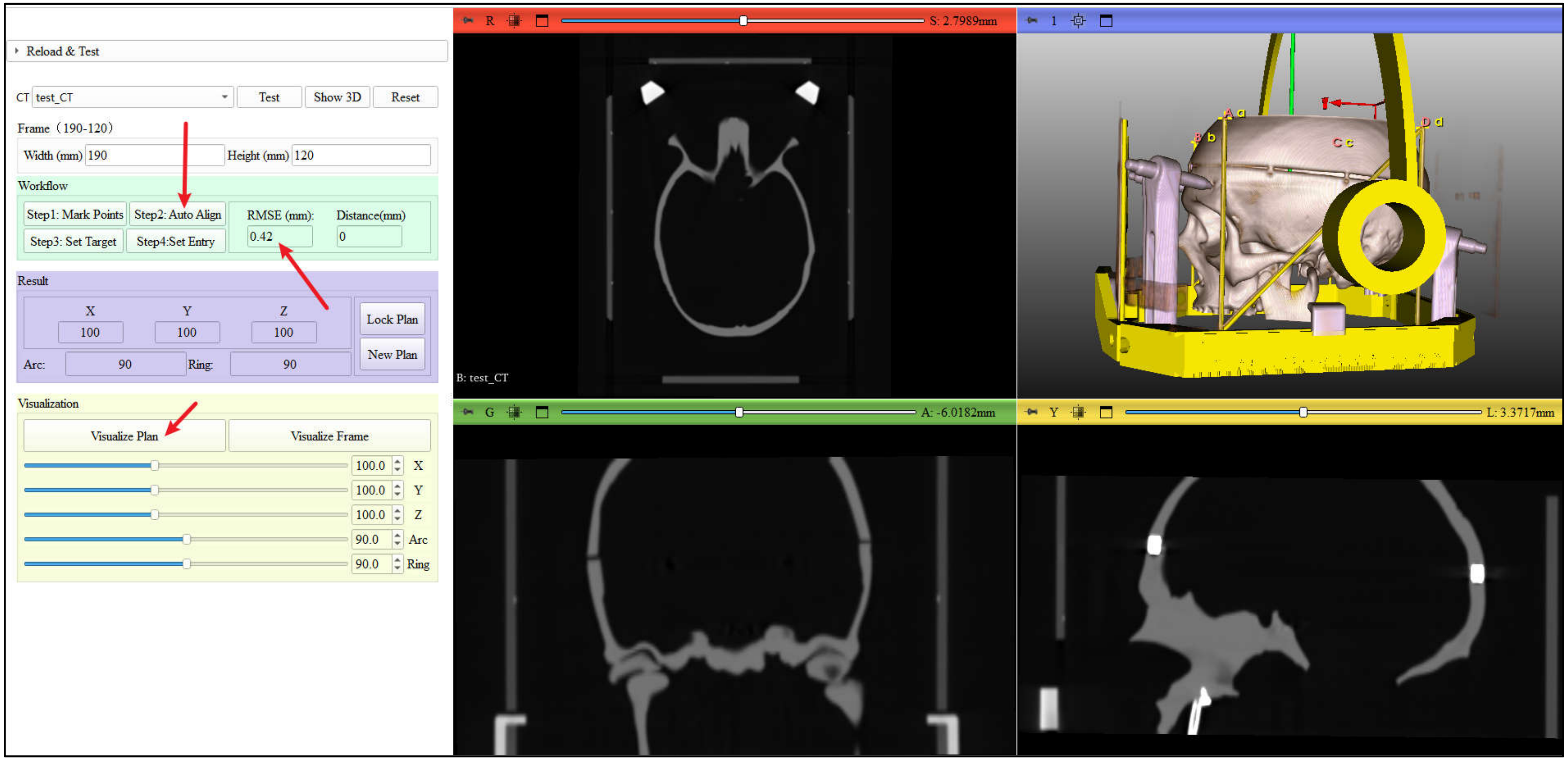

2、Module Usage

2.3 The user can adjust the contrast or slice level of the CT data to accurately identify the surgical target location. Then, click ***Step 3: Set Target*** to place the target marker, followed by placing the entry point marker in the same manner. By clicking ***Visualize Plan***, the head frame model will be synchronized to the current position. If adjustments to the target or entry point are necessary, simply drag the corresponding markers to reposition them.

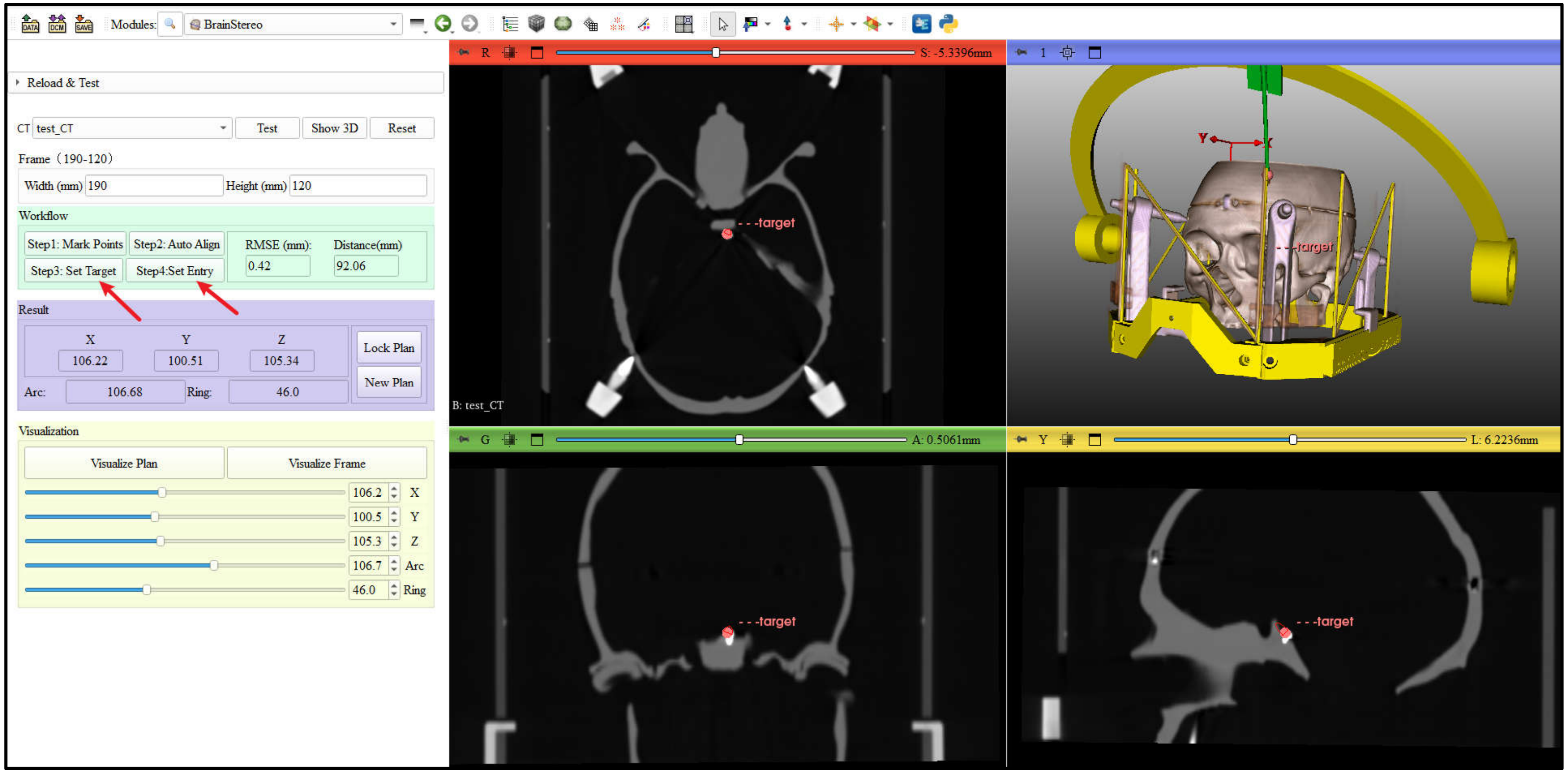

3 Other Functions

By completing the steps above, the frame registration, target and entry point selection, and result calculation can be quickly accomplished. If multiple surgical paths need to be added, after selecting a pair of target and entry points, click ***New Plan*** to place a simulated path model. Additionally, users can manually adjust the head frame penetration direction by clicking different sliders in the Visualization area, thereby simulating various surgical approaches

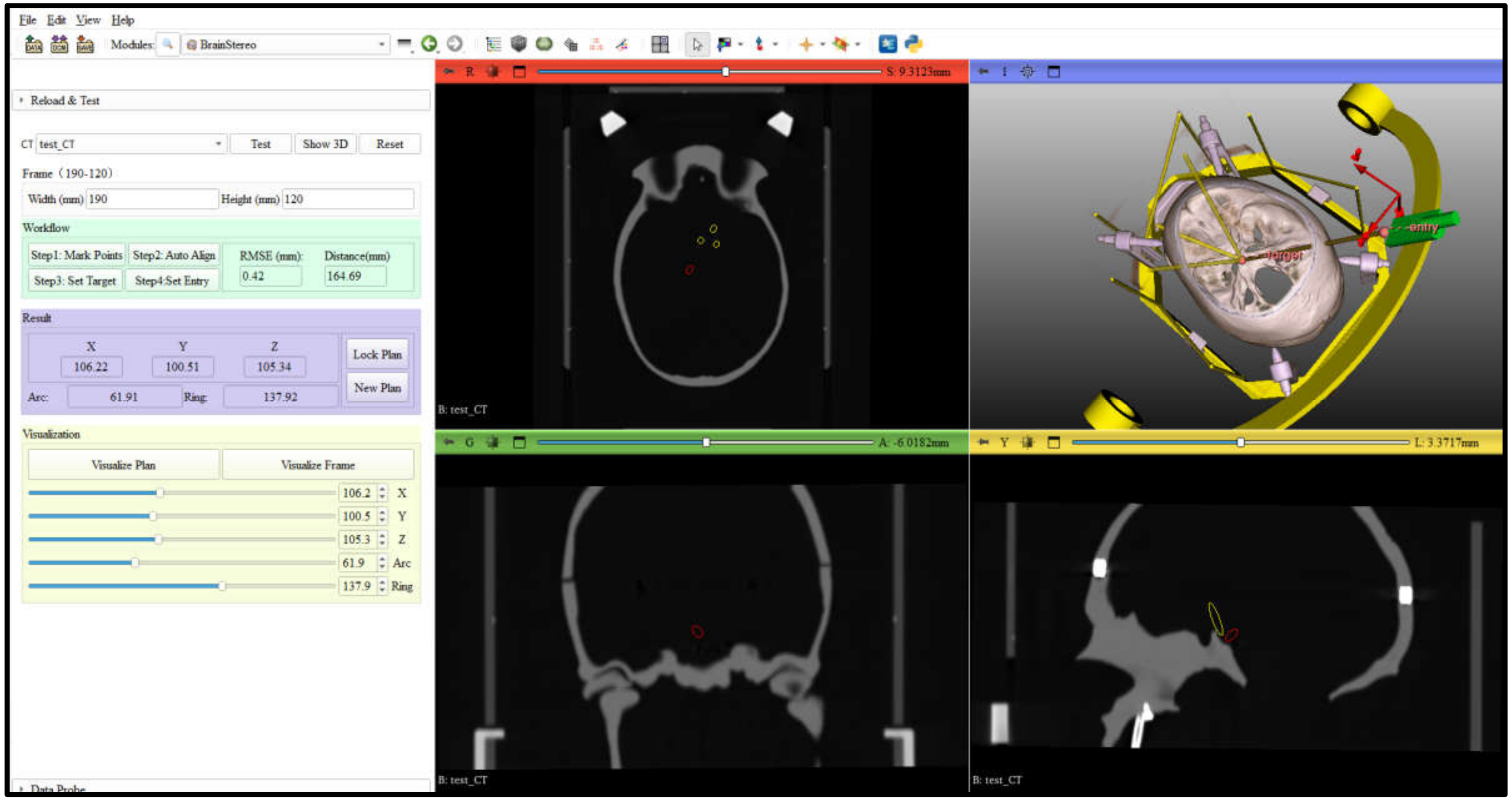

Supplement: Supplementary file 1 — Supplementary file1 (PDF 1069 KB) [file 701_2025_6564_MOESM1_ESM.pdf]
